# Supplementary material for: Dynamic diversity of SARS-CoV-2 genetic mutations in a lung transplantation patient with persistent COVID-19
Source: Nat Commun. 2024 Apr 29;15:3604. doi: 10.1038/s41467-024-47941-x (PMC11058237; doi:10.1038/s41467-024-47941-x)
Supplement: Supplementary file 1 — Supplementary Information [file 41467_2024_47941_MOESM1_ESM.pdf]

Supplementary

Table

Supplementary Table 1 Frequency of genetic and amino acid mutations in SARS-CoV-2

Nucleotide mutation	Amino acid mutation	Day 22	Day 29	Day 36	Day 62	Day 75	Day 78	Day 83	Day 86
510_518del	ORF1a:InflameDeletion	NA	NA	0.09	NA	NA	NA	NA	NA
G587A	ORF1a:V108I (nsp1:V108I)	NA	NA	NA	NA	0.05	NA	NA	NA
T670G	ORF1a:S135R (nsp1:S135R)	1	1	1	1	1	1	1	1
A752T	ORF1a:T163S (nsp1:T163S)	NA	NA	0.05	NA	NA	NA	NA	NA
C882T	ORF1a:A206V (nsp2:A26V)	NA	NA	NA	NA	0.06	NA	NA	NA
A1007C	ORF1a:S248R (nsp2:S68R)	NA	NA	NA	NA	NA	NA	NA	0.11
T1104C	ORF1a:I280T (nsp2:I100T)	NA	0.1	NA	NA	NA	NA	NA	NA
A1631G	ORF1a:K456E (nsp2:K276E)	NA	NA	0.06	NA	NA	NA	NA	NA
G2076A	ORF1a:G604D (nsp2:G424D)	NA	0.05	NA	NA	NA	NA	NA	NA
C2790T	ORF1a:T842I (nsp3:T24I)	1	1	1	1	1	1	1	1
A3126G	ORF1a:D954G (nsp3:D136G)	NA	NA	NA	NA	0.05	NA	NA	NA
G3155A	ORF1a:A964T (nsp3:A146T)	NA	NA	0.06	NA	NA	NA	NA	NA
C3290G	ORF1a:Q1009E (nsp3:Q191E)	0.03	NA	0.05	NA	NA	0.04	NA	NA
A3542G	ORF1a:T1093A (nsp3:T275A)	NA	0.04	0.04	0.06	NA	NA	NA	NA
A3637T	ORF1a:K1124N (nsp3:K306N)	NA	NA	NA	NA	NA	NA	NA	0.23
G3998A	ORF1a:E1245K (nsp3:E427K)	NA	NA	NA	NA	0.07	NA	0.04	0.23
G4184A	ORF1a:G1307S (nsp3:G489S)	1	1	1	1	1	1	1	1
C4296T	ORF1a:T1344I (nsp3:T526I)	NA	NA	0.05	NA	NA	NA	NA	NA
T4968C	ORF1a:I1568T (nsp3:I750T)	NA	NA	NA	0.1	NA	NA	NA	NA
T5119A	ORF1a:F1618L (nsp3:F800L)	0.04	NA	0.05	0.04	NA	0.04	0.04	NA
T5120A	ORF1a:Y1619N (nsp3:Y801N)	0.04	NA	0.05	0.04	NA	0.04	0.04	NA
C6636T	ORF1a:T2124I (nsp3:T1306I)	NA	NA	NA	0.91	1	1	1	1
G7498A	ORF1a:M2411I (nsp3:M1593I)	NA	NA	NA	NA	NA	NA	0.09	NA
G7791T	ORF1a:G2509V (nsp3:G1691V)	NA	NA	NA	0.14	0.19	0.07	0.42	NA
G8178A	ORF1a:R2638Q (nsp3:R1820Q)	NA	NA	NA	NA	NA	NA	NA	0.07
C8802A	ORF1a:T2846N (nsp4:T83N)	NA	NA	NA	NA	NA	NA	0.07	0.71
C9344T	ORF1a:L3027F (nsp4:L264F)	1	0.99	1	1	0.99	1	1	1
C9534T	ORF1a:T3090I (nsp4:T327I)	1	1	1	1	1	1	1	1
C10029T	ORF1a:T3255I (nsp4:T492I)	1	1	1	1	1	1	1	1
C10449A	ORF1a:P3395H (nsp5:P132H)	1	1	1	1	1	1	1	1
11075insT	ORF1a:flameshift	NA	0.03	0.06	0.04	NA	NA	0.04	NA
11075del	ORF1a:flameshift	NA	NA	NA	0.05	0.04	NA	NA	0.04
11288_11296del	ORF1a:S3675_F3677del (nsp6:S106_F108del)	0.72	0.73	0.7	0.75	0.73	0.76	0.76	0.82
C11442T	ORF1a:A3726V (nsp6:A157V)	1	1	1	1	1	1	1	1
G11743T	ORF1a:Q3826H (nsp6:Q257H)	NA	NA	NA	0.1	NA	NA	NA	NA
C12756T	ORF1a:T4164I (nsp9:T24I)	0.08	NA	NA	NA	NA	NA	NA	NA
C12789T	ORF1a:T4175I (nsp9:T35I)	NA	NA	0.11	NA	NA	NA	NA	NA

Continued

Nucleotide mutation	Amino acid mutation	Day 22	Day 29	Day 36	Day 62	Day 75	Day 78	Day 83	Day 86
T13924G	ORF1b:W153G (nsp12:W162G)	NA	NA	0.09	0.04	NA	NA	NA	NA
A13928T	ORF1b:Y154F (nsp12:Y163F)	NA	NA	0.11	0.05	NA	NA	NA	NA
A14359C	ORF1b:I298L (nsp12:I307L)	NA	NA	NA	NA	0.5	0.59	NA	NA
C14408T	ORF1b:P314L (nsp12:P323L)	1	1	1	1	1	1	1	1
G14831A	ORF1b:C455Y (nsp12:C464Y)	0.05	0.03	NA	NA	NA	NA	NA	NA
G14831T	ORF1b:C455F (nsp12:C464F)	0.05	NA	NA	NA	NA	NA	NA	NA
T14996C	ORF1b:M510T (nsp12:M519T)	NA	NA	0.06	NA	NA	NA	NA	NA
G15451A	ORF1b:G662S (nsp12:G671S)	NA	NA	NA	0.92	1	1	1	1
T15682A	ORF1b:Y739N (nsp12:Y748N)	NA	NA	0.04	0.05	NA	NA	NA	0.04
T15685A	ORF1b:L740M (nsp12:L749M)	NA	NA	0.05	0.06	NA	0.03	0.03	0.03
G15769T	ORF1b:A768S (nsp12:A777S)	0.16	NA	NA	NA	NA	NA	NA	NA
G15814A	ORF1b:V783I (nsp12:V792I)	0.26	0.31	NA	NA	NA	NA	NA	NA
G15822A	ORF1b:M785I (nsp12:M794I)	NA	NA	0.06	0.04	NA	NA	NA	NA
G15822T	ORF1b:M785I (nsp12:M794I)	NA	NA	0.51	NA	NA	NA	NA	NA
T15835C	ORF1b:C790R (nsp12:C799R)	NA	0.05	0.05	NA	NA	NA	NA	NA
G15836T	ORF1b:C790F (nsp12:C799F)	NA	0.58	0.14	0.96	1	1	1	1
T15877C	ORF1b:C804R (nsp12:C813R)	NA	NA	0.05	NA	NA	NA	NA	NA
C16289A	ORF1b:A941D (nsp13:A18D)	NA	NA	0.09	NA	NA	NA	NA	NA
C16289T	ORF1b:A941V (nsp13:A18V)	NA	NA	NA	0.92	1	1	1	1
G16411T	ORF1b:D982Y (nsp13:D59Y)	NA	NA	0.07	NA	NA	NA	NA	NA
A16412C	ORF1b:D982A (nsp13:D59A)	NA	NA	0.08	NA	NA	NA	NA	NA
T16456G	ORF1b:S997A (nsp13:S74A)	NA	NA	0.07	NA	NA	NA	NA	NA
C16604T	ORF1b:A1046V (nsp13:A123V)	NA	NA	0.06	NA	NA	NA	NA	NA
16814del	ORF1b:flameshift	NA	NA	0.05	NA	NA	NA	NA	NA
C16936T	ORF1b:P1157S (nsp13:P234S)	NA	NA	NA	NA	NA	NA	0.1	NA
T17041A	ORF1b:Y1192N (nsp13:Y269N)	NA	NA	0.06	NA	NA	NA	NA	NA
T17051C	ORF1b:V1195A (nsp13:V272A)	NA	NA	0.06	NA	NA	NA	NA	NA
T17224C	ORF1b:C1253R (nsp13:C330R)	NA	NA	0.07	NA	NA	NA	NA	NA
G17338T	ORF1b:A1291S (nsp13:A368S)	NA	NA	NA	NA	0.12	0.03	0.3	0.88
C17410T	ORF1b:R1315C (nsp13:R392C)	1	1	1	1	1	1	1	1
A18163G	ORF1b:I1566V (nsp14:I42V)	1	1	1	0.05	NA	NA	NA	NA
C18265T	ORF1b:R1600C (nsp14:R76C)	NA	NA	NA	0.92	1	1	1	1
C19955T	ORF1b:T2163I (nsp15:T112I)	1	1	1	1	1	1	1	1
G20173A	ORF1b:V2236I (nsp15:V185I)	NA	NA	NA	0.24	0.1	0.11	0.23	0.85
G20326A	ORF1b:V2287I (nsp15:V236I)	NA	NA	NA	NA	0.53	0.68	0.05	NA
21102insT	ORF1b:flameshift	0.05	NA	NA	NA	0.05	NA	NA	0.06
C21618T	S:T19I	1	1	0.99	1	1	1	1	1
21633_21641del	S:L24_P26del	0.73	0.72	0.8	0.75	0.81	0.79	0.84	0.85
G21641T	S:A27S	1	1	1	1	1	1	1	1
T21656C	S:F32L	NA	NA	NA	0.18	NA	NA	NA	NA
21765_21770del	S:H69_V70del	0.75	0.67	0.72	0.74	0.73	0.76	0.79	0.87
G21800T	S:D80Y	NA	NA	NA	NA	NA	NA	NA	0.06
G21830T	S:V90F	NA	NA	0.05	NA	NA	NA	NA	NA
G21987A	S:G142D	1	1	1	1	0.99	1	1	1
21992_21994del	S:Y144del	NA	NA	NA	0.82	0.78	0.79	0.83	0.87

Continued

Nucleotide mutation	Amino acid mutation	Day 22	Day 29	Day 36	Day 62	Day 75	Day 78	Day 83	Day 86
T22092A	S:M177K	NA	NA	NA	NA	NA	0.04	0.27	0.8
G22100A	S:E180K	NA	NA	NA	NA	0.06	0.08	NA	NA
G22103C	S:G181R	NA	NA	NA	NA	NA	0.08	NA	0.18
G22103A	S:G181R	NA	NA	NA	NA	0.52	0.65	0.07	NA
T22200G	S:V213G	1	1	1	1	1	1	1	1
G22203T	S:R214L	NA	NA	NA	0.93	1	1	1	1
C22448T	S:L296F	NA	NA	NA	NA	NA	NA	0.05	NA
C22550T	S:P330S	NA	NA	0.14	NA	NA	NA	NA	NA
G22578A	S:G339D	1	1	1	1	1	1	1	1
G22599C	S:R346T	NA	NA	NA	1	1	1	1	1
T22667C	S:Y369H	NA	NA	0.1	NA	NA	NA	NA	NA
C22674T	S:S371F	1	1	1	0.99	1	1	1	1
T22679C	S:S373P	1	0.99	1	1	1	1	1	1
C22686T	S:S375F	1	0.99	0.99	1	1	1	0.99	1
A22688G	S:T376A	1	1	0.99	1	1	1	1	1
A22728G	S:D389G	NA	NA	NA	0.49	0.85	0.97	0.73	0.16
G22775A	S:D405N	0.98	0.89	0.95	1	1	1	1	1
A22786C	S:R408S	0.93	0.93	0.93	0.93	0.94	0.93	0.92	0.93
G22813T	S:K417N	1	1	1	1	0.99	1	1	1
T22882G	S:N440K	1	1	0.99	1	1	1	1	1
T22917G	S:L452R	1	1	1	0.99	0.99	1	0.99	1
G22992A	S:S477N	1	1	1	1	1	1	1	1
C22995A	S:T478K	1	1	1	1	1	1	1	1
A23013C	S:E484A	1	1	1	1	1	1	1	0.99
T23018G	S:F486V	1	1	0.98	0.05	NA	NA	NA	NA
A23055G	S:Q498R	1	1	1	1	1	0.99	0.99	1
A23063T	S:N501Y	1	0.99	1	1	1	1	1	1
G23072A	S:G504S	NA	NA	0.06	NA	NA	NA	NA	NA
G23073A	S:G504D	NA	0.31	NA	NA	NA	NA	NA	NA
T23075C	S:Y505H	1	0.99	1	0.99	1	0.99	1	1
C23202A	S:T547K	NA	NA	NA	NA	0.15	NA	0.26	0.82
A23403G	S:D614G	0.99	1	0.99	1	0.99	1	1	1
G23405T	S:V615F	NA	NA	NA	NA	NA	NA	0.07	NA
G23463T	S:R634L	NA	NA	NA	0.23	0.16	NA	0.27	0.83
G23463A	S:R634H	NA	NA	NA	0.1	NA	NA	NA	NA
T23487G	S:V642G	NA	NA	NA	0.46	0.82	0.97	0.74	0.17
C23525T	S:H655Y	1	1	1	0.99	1	1	1	1
C23589A	S:T676N	NA	NA	NA	0.92	1	1	1	1
A23592C	S:Q677P	NA	NA	NA	0.08	NA	NA	NA	NA
A23598G	S:N679S	NA	NA	NA	NA	NA	NA	NA	0.16
T23599G	S:N679K	1	1	1	0.99	1	1	1	1
C23604A	S:P681H	1	1	1	1	1	1	1	1
C23854A	S:N764K	1	1	1	1	1	1	1	1
G23948T	S:D796Y	1	1	1	1	0.99	1	1	1
C24117T	S:A852V	NA	NA	NA	0.08	NA	NA	0.28	0.9

Continued

Nucleotide mutation	Amino acid mutation	Day 22	Day 29	Day 36	Day 62	Day 75	Day 78	Day 83	Day 86
A24424T	S:Q954H	1	1	1	1	0.99	1	1	1
T24469A	S:N969K	1	1	1	1	1	1	0.99	1
G25145A	S:E1195K	NA	NA	NA	0.94	1	1	1	1
A25325T	S:K1255*	NA	NA	0.06	0.05	NA	NA	NA	NA
G25352C	S:V1264L	NA	NA	NA	NA	NA	NA	0.05	NA
G25690C	ORF3a:G100R	NA	NA	NA	NA	NA	NA	0.05	NA
C26060T	ORF3a:T223I	1	1	1	1	1	1	1	1
C26270T	E:T9I	1	1	1	1	1	1	1	1
G26529A	M:D3N	1	1	1	1	1	1	1	1
C26577G	M:Q19E	0.99	1	1	1	1	1	1	1
G26709A	M:A63T	1	1	1	1	1	1	1	1
T27242C	ORF6:I14T	NA	NA	NA	NA	NA	NA	0.05	NA
G27463A	ORF7a:V24I	NA	0.39	NA	NA	NA	NA	NA	NA
C27999T	ORF8:P36S	NA	NA	NA	NA	NA	NA	0.69	NA
C28087A	ORF8:A65D	NA	NA	NA	NA	0.6	0.87	0.04	NA
C28311T	N:P13L	1	1	1	1	1	1	1	1
28362_28370del	N:E31_S32del	0.68	0.71	0.7	0.71	0.7	0.74	0.77	0.86
G28881A	N:R203K	1	1	1	1	1	1	1	1
G28883C	N:G204R	1	1	1	1	1	1	1	1
A29039T	N:K256*	0.04	0.05	NA	0.05	0.05	0.08	NA	0.03
C29085T	N:T271I	NA	NA	NA	NA	NA	NA	0.06	0.78
G29384C	N:D371H	NA	NA	0.03	0.08	0.08	0.06	0.05	0.11
A29510C	N:S413R	1	1	1	1	1	1	1	1

In this table, all mutations with a frequency of 5% or higher as determined using whole genome sequencing are listed.

NA: Not applicable

Supplementary Table 2 Primers used for the SARS-CoV-2 whole-genome sequencing

Name	Sequence	Region	Pool
Sc2Wgs2kb_1_LEFT	5′-ACCAACCAACTTTCGATCTCTTGT-3′	30–54	1
Sc2Wgs2kb_1_RIGHT	5′-CCAATCAAGGACGGGTTTGAGT-3′	2133–2155	1
Sc2Wgs2kb_2_LEFT	5′-TCACTGAGACTCATTGATGCTATGA-3′	1993–2018	2
Sc2Wgs2kb_2_RIGHT	5′-ACCCTCTTGAACAACATCACCC-3′	4125–4147	2
Sc2Wgs2kb_3_LEFT	5′-TGGAAGAAACTAAGTTCCTCACAGA-3′	3992–4017	3&4
Sc2Wgs2kb_3_RIGHT	5′-ATCAATAGCCACCACATCACCA-3′	6156–6178	3&4
Sc2Wgs2kb_4_LEFT	5′-CCAACCATATCCAAACGCAAGC-3′	6015–6037	1
Sc2Wgs2kb_4_RIGHT	5′-GCAAGTTCAGCTTCTGCAGTTG-3′	8105–8127	1
Sc2Wgs2kb_5_LEFT	5′-TGATAGTGCGGAAGTTGCAGTT-3′	8007–8029	2
Sc2Wgs2kb_5_RIGHT	5′-ACCCTCAACTTTACCAGATGGG-3′	10077–10099	2
Sc2Wgs2kb_6_LEFT	5′-TCTCGCAAAGGCTCTCAATGAC-3′	9963–9985	3
Sc2Wgs2kb_6_RIGHT	5′-AGCTTGTAAGGTTGCCCTGTTG-3′	12072–12094	3
Sc2Wgs2kb_7_LEFT	5′-GGGCTCAATGTGTCCAGTTACA-3′	11927–11949	1
Sc2Wgs2kb_7_RIGHT	5′-CAATACCAGCATTTCGCATGGC-3′	14022–14044	1
Sc2Wgs2kb_8_LEFT	5′-GGACTGGTATGATTTTGTAGAAAACCC-3′	13919–13946	2
Sc2Wgs2kb_8_RIGHT	5′-AGCTAAAGACACGAACCGTTCA-3′	16007–16029	2
Sc2Wgs2kb_9_LEFT	5′-AGGACCTCATGAATTTTGCTCTCA-3′	15860–15884	3
Sc2Wgs2kb_9_RIGHT	5′-CCTACGTGGAATTTCAAGACTTGT-3′	17997–18021	3
Sc2Wgs2kb_10_LEFT	5′-ACCACTGAAACAGCTCACTCTT-3′	17880–17902	1
Sc2Wgs2kb_10_RIGHT	5′-AGTGAGTGGTGCACAAATCGTT-3′	19958–19980	1
Sc2Wgs2kb_11_LEFT	5′-GGGTGTGGACATTGCTGCTAAT-3′	19844–19866	2
Sc2Wgs2kb_11_RIGHT	5′-CAATAAGTAGGGACTGGGTCTTCG-3′	21896–21920	2
Sc2Wgs2kb_12_LEFT	5′-TGTCTCTGGGACCAATGGTACT-3′	21768–21790	3&4
Sc2Wgs2kb_12_RIGHT	5′-TGTGCAAAAACTTCTTGGGTGT-3′	23891–23913	3&4
Sc2Wgs2kb_13_LEFT	5′-TGTGGTGATTCAACTGAATGCAG-3′	23788–23811	1
Sc2Wgs2kb_13_RIGHT	5′-AGTCGTAACAATTAGTATGCCAGCA-3′	25833–25858	1
Sc2Wgs2kb_14_LEFT	5′-GCCTTGAAGCCCCTTTTCTCTA-3′	25690–25712	2
Sc2Wgs2kb_14_RIGHT	5′-AGCAGAAAGGCTAAAAAGCACAA-3′	27785–27808	2
Sc2Wgs2kb_15_LEFT	5′-ACTGTTCATCAGACAAGAGGAAGT-3′	27647–27671	3
Sc2Wgs2kb_15_RIGHT	5′-ATAGGCAGCTCTCCCTAGCATT-3′	29769–29791	3

Figure

Supplementary Figure 1 Number of amino acid mutations observed during the second and third round of RDV administration.

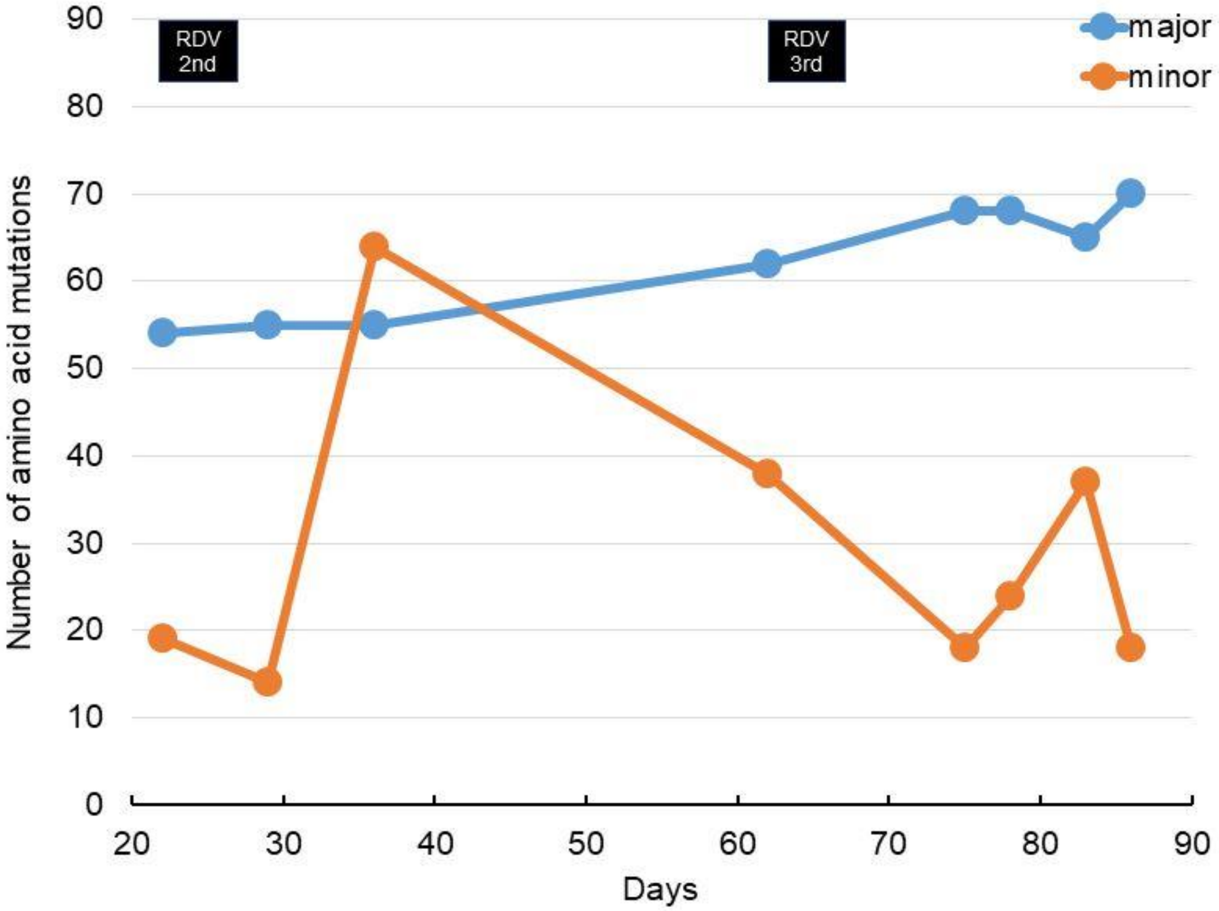

The amino acid mutations compared with those in the index strain of SARS-CoV-2 (NC_045512.2, Pango lineage B) were categorized as major and minor based on a frequency of more and less than 50%, respectively. All mutations with a frequency of 5% or higher are shown.

The number of minor mutations reached a maximum of 64 on Day 36 and decreased thereafter. It should be noted that minor mutations were observed to increase within two to three weeks of the start of both the second and third RDV administration. Most of the minor mutations were thereafter eliminated, but some, such as C799F, persisted to become major mutations.

RDV: Remdesivir

Supplementary Figure 2 Comparison of RDV sensitivity of CH-LT4 and later isolates with that of CH-LT1

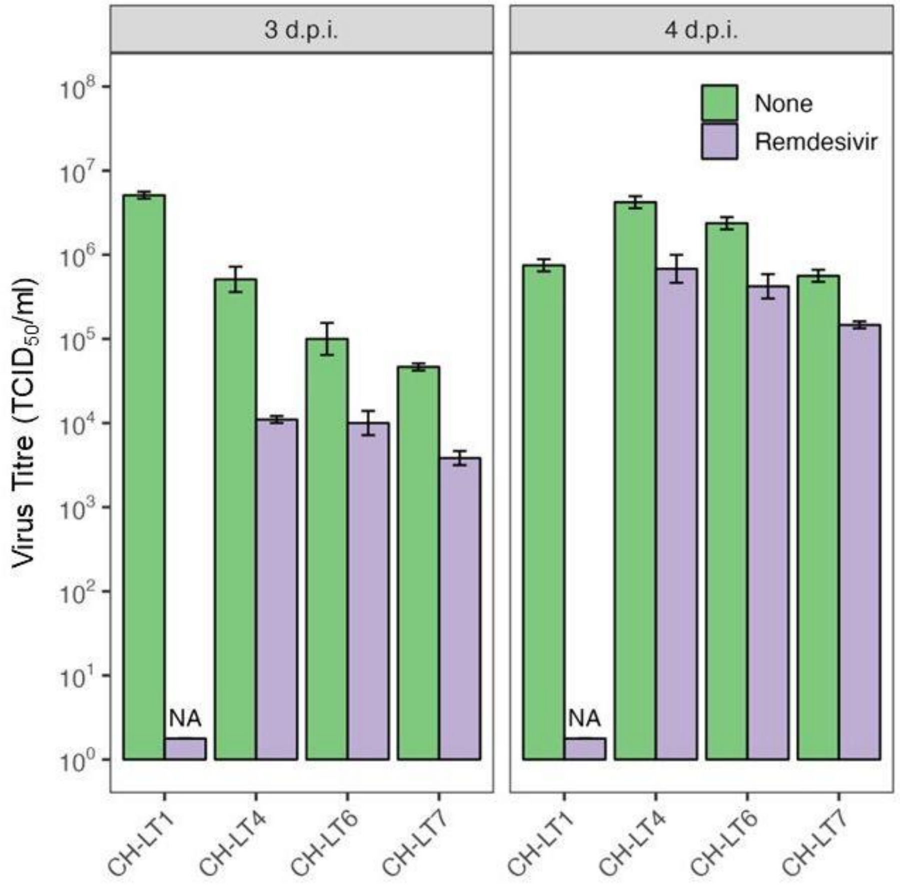

To examine whether CH-LT4 and later isolates still possess the RDV resistance or not, we performed the comparison experiments of the later isolates with the original isolate CH-LT1 in the presence or absence of 5μM RDV at two fixed points: 3 and 4 d.p.i. CH-LT4 and later isolates could propagate at least to some extent (approximately one order) in the presence of 5μM RDV whereas CH-LT1 was suppressed to undetectable level. NA means not applicable (detection limit was 10 $TCID_{50}$/mL). Error bars indicate standard errors (SE).

RDV: Remdesivir

Supplementary Figure 3 Chemical structures of RDV-TP and ATP

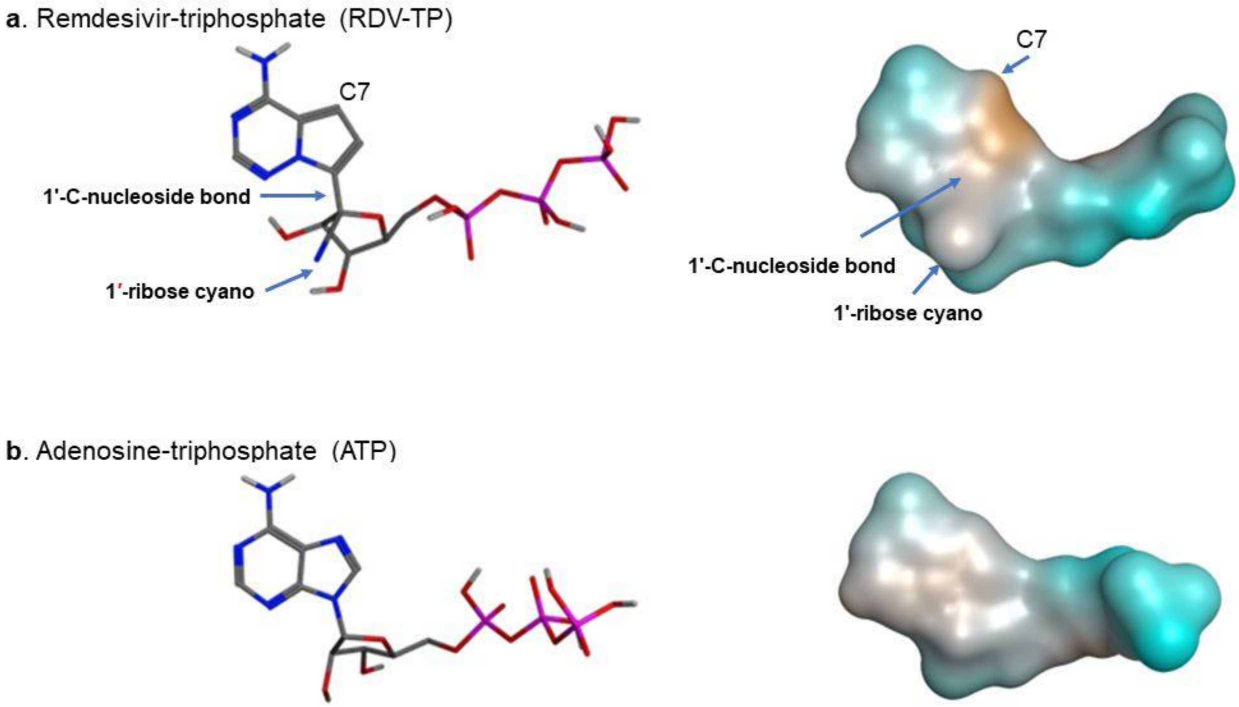

a. Remdesivir-triphosphate (RDV-TP).

b. Adenosine-triphosphate (ATP).

Left: stick model, Right: Space-filling model, Cyan: hydrophilic, Orange: hydrophobic.

Supplementary information

The iVar commands for whole genome sequencing

Preprocessing with fastp

fastp -i read1.fastq.gz -I read2.fastq.gz -o read1.fastp.fastq.gz -O read2.fastp.fastq.gz -f 1 -F 1 -t 1 -T 1

Mapping to a reference genome with BWA

bwa mem NC_045512.fasta read1.fastp.fastq.gz read2.fastp.fastq.gz | samtools view -b -q 1 | samtools sort -o sample.sorted.bam

samtools index sample.sorted.bam

Trim primer sequences with iVar

ivar trim -b PrimerRegion.bed -p sample.sorted.trimmed.bam -i sample.sorted.bam -e

samtools sort -o sample.sorted.trimmed.sorted.bam sample.sorted.trimmed.bam

samtools index sample.sorted.trimmed.sorted.bam

Call variants with iVar

samtools mpileup -B -q 1 sample.sorted.trimmed.sorted.bam | ivar variants -p iVarVariants -q 20 -t 0.03 -r NC_045512.fasta -g NC_045512.gff

Generate a consensus sequence with iVar

samtools mpileup -B -q 1 sample.sorted.trimmed.sorted.bam | ivar consensus -p ConsensusSeq -q 20 -t 0

The commands for phylogenetic analysis

Multiple sequence alignment by MAFFT

mafft --auto MultiFasta.fasta > output.aln

Inferring phylogenetic tree by IQ-TREE

iqtree2 -s output.aln -m MFP -bb 1000
