# Supplementary material for: Dynamic diversity of SARS-CoV-2 genetic mutations in a lung transplantation patient with persistent COVID-19
Source: Nat Commun. 2024 Apr 29;15:3604. doi: 10.1038/s41467-024-47941-x (PMC11058237; doi:10.1038/s41467-024-47941-x)
Supplement: Supplementary file 3 — Reporting Summary [file 41467_2024_47941_MOESM3_ESM.pdf]

nature portfolio

Corresponding author(s): Hidetoshi Igari, Takayuki Ishige, Eiji Ido

Last updated by author(s): Apr 12, 2024

Reporting Summary

Nature Portfolio wishes to improve the reproducibility of the work that we publish. This form provides structure for consistency and transparency in reporting. For further information on Nature Portfolio policies, see our Editorial Policies and the Editorial Policy Checklist.

Statistics

For all statistical analyses, confirm that the following items are present in the figure legend, table legend, main text, or Methods section.

n/a	Confirmed	
☒	☐	The exact sample size (*n*) for each experimental group/condition, given as a discrete number and unit of measurement
☐	☒	A statement on whether measurements were taken from distinct samples or whether the same sample was measured repeatedly
☒	☐	The statistical test(s) used AND whether they are one- or two-sided<br>*Only common tests should be described solely by name; describe more complex techniques in the Methods section.*
☒	☐	A description of all covariates tested
☒	☐	A description of any assumptions or corrections, such as tests of normality and adjustment for multiple comparisons
☐	☒	A full description of the statistical parameters including central tendency (e.g. means) or other basic estimates (e.g. regression coefficient) AND variation (e.g. standard deviation) or associated estimates of uncertainty (e.g. confidence intervals)
☒	☐	For null hypothesis testing, the test statistic (e.g. *F*, *t*, *r*) with confidence intervals, effect sizes, degrees of freedom and *P* value noted<br>*Give P values as exact values whenever suitable.*
☒	☐	For Bayesian analysis, information on the choice of priors and Markov chain Monte Carlo settings
☒	☐	For hierarchical and complex designs, identification of the appropriate level for tests and full reporting of outcomes
☒	☐	Estimates of effect sizes (e.g. Cohen's *d*, Pearson's *r*), indicating how they were calculated

Our web collection on statistics for biologists contains articles on many of the points above.

Software and code

Policy information about availability of computer code

Data collection	No software was used for data collection
Data analysis	Whole-genome sequencing<br>fastp (version 0.23.2, https://github.com/OpenGene/fastp)<br>bwa (version: 0.7.17-r1188, https://github.com/lh3/bwa)<br>iVar (version 1.4, https://andersen-lab.github.io/ivar/html/index.html)<br>Phylogenetic analysis<br>MAFFT (version 7, https://mafft.cbrc.jp/alignment/software/)<br>IQ-TREE (version 2.2.6, http://www.iqtree.org)<br>iTOL (version 6.8.1, https://itol.embl.de)<br>Dose-response analysis for RDV resistance experiment<br>R (version 4.2.2, https://www.r-project.org)<br>drc package (version 3.0-1, https://cran.r-project.org/web/packages/drc/index.html)<br>Three-dimensional structure analysis<br>Molecular Operating Environment (MOE), version 2016.08 (CCG Inc, Montreal, Canada) is commercially available. (https://www.chemcomp.com/index.htm)

For manuscripts utilizing custom algorithms or software that are central to the research but not yet described in published literature, software must be made available to editors and reviewers. We strongly encourage code deposition in a community repository (e.g. GitHub). See the Nature Portfolio guidelines for submitting code & software for further information.

Data

Policy information about availability of data

All manuscripts must include a data availability statement. This statement should provide the following information, where applicable:

- Accession codes, unique identifiers, or web links for publicly available datasets
- A description of any restrictions on data availability
- For clinical datasets or third party data, please ensure that the statement adheres to our policy

Consensus sequence data are available from GISAID: the accession numbers, EPI_ISL_18261614–EPI_ISL_18261621. In addition, fastq files were also uploaded to NCBI Sequence Read Archive: BioProject number, PRJNA983865 (BioSample accession numbers, SAMN35736960–SAMN35736967).
All the software(s) used in our study are freely available through the following sites except Molecular Operating Environment (MOE).

Whole-genome sequencing
fastp (version 0.23.2, https://github.com/OpenGene/fastp)
bwa (version: 0.7.17-r1188, https://github.com/lh3/bwa)
iVar (version 1.4, https://andersen-lab.github.io/ivar/html/index.html)

Phylogenetic analysis
MAFFT (version 7, https://mafft.cbrc.jp/alignment/software/)
IQ-TREE (version 2.2.6, http://www.iqtree.org)
iTOL (version 6.8.1, https://itol.embl.de)

Dose-response analysis for RDV resistance experiment
R (version 4.2.2, https://www.r-project.org)
drc package (version 3.0-1, https://cran.r-project.org/web/packages/drc/index.html)

Molecular Operating Environment (MOE), version 2016.08 (CCG Inc, Montreal, Canada) is commercially available. (https://www.chemcomp.com/index.htm)

Research involving human participants, their data, or biological material

Policy information about studies with human participants or human data. See also policy information about sex, gender (identity/presentation), and sexual orientation and race, ethnicity and racism.

Reporting on sex and gender	We do not write for avoiding identifying the person.
Reporting on race, ethnicity, or other socially relevant groupings	We do not write for avoiding identifying the person.
Population characteristics	A person at the age of 50s after lung transplantation
Recruitment	This case was a chronic persistent infection of COVID-19 after lung transplantation and his virus revealed a dynamic genetic diversity with many amino acid mutations involving remdesivir resistance in a clinical course. Therefore we decided to report as a case report with various analytic data.
Ethics oversight	The Research Ethics Committees of Graduate School of Medicine, Chiba University

Note that full information on the approval of the study protocol must also be provided in the manuscript.

Field-specific reporting

Please select the one below that is the best fit for your research. If you are not sure, read the appropriate sections before making your selection.

- [x] Life sciences
- [ ] Behavioural & social sciences
- [ ] Ecological, evolutionary & environmental sciences

For a reference copy of the document with all sections, see nature.com/documents/nr-reporting-summary-flat.pdf

Life sciences study design

All studies must disclose on these points even when the disclosure is negative.

Sample size	One. Because this paper is a case report of chronic persistent infection of COVID-19 after lung transplantation.
Data exclusions	No. This study is a case report. Therefore there were no cases of exclusion.
Replication	All the assays to confirm SARS-CoV-2 pathogenicity, SARS-CoV-2 growth kinetics and remdesivir resistance were conducted three times. The whole genome sequence of every isolated SARS-CoV-2 was conducted one time.

Randomization	No. This study is a case report. Therefore there was no randomization.
Blinding	This study is a case report. Therefore Information regarding the clinical course was shared among all the team members.

Reporting for specific materials, systems and methods

We require information from authors about some types of materials, experimental systems and methods used in many studies. Here, indicate whether each material, system or method listed is relevant to your study. If you are not sure if a list item applies to your research, read the appropriate section before selecting a response.

Materials & experimental systems

n/a	Involved in the study
x	Antibodies
	x Eukaryotic cell lines
x	Palaeontology and archaeology
x	Animals and other organisms
	x Clinical data
x	Dual use research of concern
x	Plants

Methods

n/a	Involved in the study
x	ChIP-seq
x	Flow cytometry
x	MRI-based neuroimaging

Eukaryotic cell lines

Policy information about cell lines and Sex and Gender in Research

Cell line source(s)	JCRB Cell Bank (Japanese Collection of Research Bioresources Cell Bank, Tokyo, Japan)
Authentication	The use of this cell line [VeroE6/TMPRSS2 (JCRB1819)] was authorized by above-mentioned organization under the name of co-author Kengo Saito.
Mycoplasma contamination	We confirmed that the result of Mycoplasma contamination was negative.
Commonly misidentified lines (See ICLAC register)	None

Clinical data

Policy information about clinical studies

All manuscripts should comply with the ICMJEguidelines for publication of clinical research and a completedCONSORT checklist must be included with all submissions.

Clinical trial registration	n/a
Study protocol	This study was approved by the Research Ethics Committees of Graduate School of Medicine, Chiba University (M10505).
Data collection	Clinical samples were collected from July 2022 to Oct 2022.
Outcomes	Description of clinical course and mutational analysis of SARS-CoV-2 together with its phenotypes

Plants

Seed stocks	*Report on the source of all seed stocks or other plant material used. If applicable, state the seed stock centre and catalogue number. If plant specimens were collected from the field, describe the collection location, date and sampling procedures.*
Novel plant genotypes	*Describe the methods by which all novel plant genotypes were produced. This includes those generated by transgenic approaches, gene editing, chemical/radiation-based mutagenesis and hybridization. For transgenic lines, describe the transformation method, the number of independent lines analyzed and the generation upon which experiments were performed. For gene-edited lines, describe the editor used, the endogenous sequence targeted for editing, the targeting guide RNA sequence (if applicable) and how the editor was applied.*
Authentication	*Describe any authentication procedures for each seed stock used or novel genotype generated. Describe any experiments used to assess the effect of a mutation and, where applicable, how potential secondary effects (e.g. second site T-DNA insertions, mosiacism, off-target gene editing) were examined.*
